# Investigation of the embryotoxic and teratogenic effect of Hypericum perforatum in pregnant rats

**DOI:** 10.4274/tjod.84429

**Published:** 2018-06-21

**Authors:** Fadime Kahyaoğlu, Alpaslan Gökçimen, Buket Demirci

**Affiliations:** 1 Avrasya University Vocational School of Health Services, Department of Pathology Laboratory Techniques, Trabzon, Turkey; 2 Adnan Menderes University Faculty of Medicine, Department of Histology and Embryology, Aydın, Turkey; 3 Adnan Menderes University Faculty of Medicine, Department of Medical Pharmacology, Aydın, Turkey

**Keywords:** Pregnancy, rat, St. Johns Worth, teratogenity

## Abstract

**Objective:**

*Hypericum perforatum* (HP) is a herbal product used in the treatment of depression, but its harm on the fetus has not been established. This study investigated the effects of HP according to fetal clinical, morphologic, and histologic findings. Study design is an animal study.

**Materials and Methods:**

Fifty-four 4-5-month-old female Wistar rats were divided into three groups: control, 100 mg/kg HP, and 300 mg/kg HP. HP treatment using drinking water was started one week before mating and ended with the delivery of pups.

**Results:**

HP exposure before conception diminished the pregnancy rate and decreased the fetal number; during pregnancy it tended to increase the duration of gestation, and deteriorated the fetal development as determined using body weight. It also damaged liver and kidney tissues, most probably due to oxidative stress, as supported through inducible nitric oxide synthase antibody staining findings at both doses.

**Conclusion:**

HP should not be recommended to women who would like to be pregnant or are pregnant because it can be harmful for both fetal and maternal health.

**PRECIS:** Considering of required studies about Hypericum perforatum (HP) and fetal exposure, this study has been evaluated adverse effects of HP on fetus both with the clinical findings and histopathological assessment of fetal liver and kidney.

## Introduction

*Hypericum perforatum* (HP), of the Clusiaceae (Hypericeae=Guttiferae) family, belongs to the subfamily Hypericoideae^(1,2,3,4)^. HP contains several groups of components that contribute to its pharmacologic activity. These are naphthodianthrones (hypericin, pseudohypercin), phloroglucinols (hyperforin, adipherforin), flavonoids (rutin, hyperoside, quercitrin) xanthones and tannins. HP has recently received interest as a herbal product that has anti-inflammatory and antiviral properties, and is effective for wound healing, inflammatory bowel disease, and depression^(1,5)^. Depression is an important disease that affects the whole society at all ages and the incidence in pregnancy period is reported as 18-19%^(6)^. It is known that the antidepressant activity of the plant (at doses of 900 mg/kg) is related to hypericin and its derivatives. It can also be abused for believing well-being and well-feeling (Biggs et. al.^(7)^ 2017). There are no well-established controlled clinical trials evaluating the safety of HP use, even in the European Medical Agency Guidelines (EMA/HMPC/244315/2016, 2018),^(8)^ for patients who want to become pregnant or are pregnant while under HP treatment. The view that “herbal products are less harmful” is misleading; their use without rigorous research can also bring about important health issues.

Therefore, we aimed to investigate the embriotoxic/teratogenic effects of HP according to fetal clinical, morphologic, and histologic findings.

## Materials and Methods

Fifty-four 4-5-month-old female Wistar rats were obtained from the experimental animal center of our university and all tests were conducted according to the principles and guidelines of the university animal ethical committee’s approval (HADYEK 2015/67). HP was obtained from local pharmacy store (St. John’s Wort Herb Extract/SOLGAR İstanbul, Turkey). On the study day, the rats were randomly assigned to three groups of 18 animals. Control group: This group of rats has been taken with water freely and served as control.

Low dose HP group: 100 mg/kg HP given to the rats with drinking water, which was available ad libitum.

High dose HP group: 300 mg/kg HP given to the rats with drinking water, which was available ad libitum.

HP treatment was started one week before mating, similar to the Gregoretti et al.^(9)^ study, and continued till delivery. The rats were weighed every Monday to adjust the HP doses. The calculated amount of HP was mixed with drinking water every morning, making sure that there was no remnants from the previous day. The suggested daily dose of HP is 900 mg (or 15 mg/kg per day for a 60-kg person) for humans; Rayburn et al.^(10)^, calculated the rodent dose as 180 mg/kg per day in their study. Gregoretti et al.^(9)^ calculated the surface area of rats and determined the dose as 100 and 1000 mg/kg for rats. Given that our aim was not to work with high doses, rather just to mimic real life, considering Rayburn et al.’s^(10)^ study, we administered two different doses of HP treatment to gain a better understanding of dose effect and decided upon 100 mg/kg and 300 mg/kg.

Maternal rats were sacrificed under general anesthesia of ketamine and xylazine (50 and 5 mg/kg, respectively) immediately after delivery. Obtained offspring were decerebrated, then morphologically examined and fixed in formalin solution. The obtained preparations were evaluated using hematoxylin and eosin and immunohistochemical staining.

### Statistical Analysis

All biologic parameters were assessed using the Mann-Whitney U test (IBM SPSS Statistics for Windows; Version 19.0, IBM Corp., NY, US).

## Results

### Morphologic-clinical assessment

The number of fetuses was six in the low-dose (100 mg/kg) group, three in the high-dose (300 mg/kg) group, and eight in the control group; the pregnancy rate decreased in a dose-dependent manner (Table 1). In the high-dose treatment group there was also a tendency for delayed delivery; more offspring were born on day 22 (Table 1). The total number of pups also decreased (Table 2); the difference was statistically significant (p=0.014). No structural extremity anomalies, facial anomalies or differences of eye openness were observed in any pups. Regarding the weight and length of the fetuses, there was a 19.9% reduction in the weight of the fetuses of the low-dose group, and an 8.4% reduction in the high-dose group (Table 3).

### Hematoxylin and eosin assessment

Our histologic evaluation showed an inflammatory reaction in the liver of the offspring of both treated groups. Additionally, focal necrosis was detected in each lobe, deteriorating cell layout at 300 mg/kg. Hydropic and vacuolar degeneration was also observed in the fetuses of rats with high-dose HP treatment. No fatty change of the liver was found. Hematopoiesis was not disrupted and continued in the fetal period.

**Figure 1:**
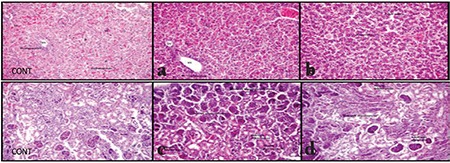
Hematoxylin and eosin (X20) staining of control group and treated individuals. a) Liver tissue of rat embryo treated with low dose 100 mg/kg *Hypericum perforatum* (HP) treatment, b) Liver tissue of rat embryo treated with high-dose 300 mg/kg HP treatment, c) Kidney tissue of rat embryo treated with low dose 100 mg/kg HP treatment, d) Renal tissue of rat embryo treated with high-dose 300 mg/kg HP treatment

In the kidney tissues, we found that the diameter of glomeruli was decreased, the Bowman capsule distance was absent and intense congestion was observed equally in the offspring of both plant-treated groups. Additionally, hydropic and hyaline degeneration was seen in kidney tubules (Figure 1).

### Immunohistochemical assessment

**Figure 2:**
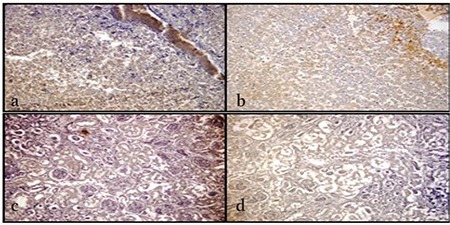
Inducible isoform immunohistochemistry (X20) staining for treated individuals. a) Liver tissue of rat embryo treated with low dose 100 mg/kg *Hypericum perforatum* (HP) treatment, b) Liver tissue of rat embryo treated with high-dose 300 mg/kg HP treatment, c) Kidney tissue of rat embryo treated with low dose 100 mg/kg HP treatment, d) Renal tissue of rat embryo treated with high-dose 300 mg/kg HP treatment

Inducible nitric oxide synthase (iNOS) antibody staining of the tissues was used to determine oxidative stress parameters. Levels of damage were determined in fetal liver tissues of rats as low (++) and high (+++). When evaluating oxidative damage in kidney tissues of the fetuses, the damage was determined as low (++) and high (+++) (Figure 2).

## Discussion

As in all herbal medicines, HP is considered innocuous and widely used against depression, and even women who are pregnant or lactating are also exposed to HP^(6)^. However, the effects of its use on gestation have yet to be clarified^(8)^.

Limited numbers of experimental studies of HP are available; different results have been obtained in animal experiments. In one study, 36 mg/kg/day was given to 15 rats in the organogenesis period (days 9-15), they were sacrificed on the 21^st^ day of pregnancy, and the number of fetuses and resorption rates were calculated during a laparotomy. The size of the fetuses was also measured and the result of the clinical examination showed that HP was not embryo toxic. Given that the fluid and food intake and weight change of the animals were the same as those of the control rats, HP was not found as toxic for the mothers either, but a histologic examination was not included in this study^(11)^. That study is similar to our work in some direction; no macroscopic difference was determined in the offspring. However, unlike our study, we also found that the rate of conception decreased dose-dependently by giving HP one week before mating. Additionally, the high-dose treatment groups’ delivery day tended to delay. Previously, the Calcium channel antagonist properties of HP were shown on rat aorta; in this case, HP might also behave as a dose-dependent tocolytic agent^(4)^. On the other hand, the conception rate and the number of pups also decreased. This point needs further investigation regarding the ovarian capacity effect of HP. At some point, 100 mg/kg or 300 mg/kg HP was enough to cause a detrimental effect on the gestation rate, gestation duration, and offspring number. Regarding the weight and length of the fetuses, there was a 19.9% and 8.4% reduction in the weight of the fetuses. Only the findings of Rayburn et al.,^(12)^ support our findings; they also found birth weights of male mice were less than controls. Two of Rayburn et al.’s^(12)^ studies were on cognitive-behavioral changes and the authors reported its safety with regards cognitive functions, but a toxicity and histopathologic evaluation on pups tissues were not performed^(10)^. In a study, HP was started on the 3^rd^ gestational day and ended on the 21^st^ postnatal day. The authors found no effect on the duration of gestation or offspring body weight alteration, but they described some treated groups weighed significantly less than the controls on the 56^th^ postnatal day. As a result, HP was found to affect the development of mice without seriously affecting their neurobehavioral development^(13)^. Chan et al.^(14)^ concluded that giving the active component, hypericin (14.2 and 142.0 ng/mL), to embryo cultures was teratogenic on rat embryos.

In another study, HP was administered via gavage to rats at two different doses, 100 mg/kg and 1000 mg/kg, starting 2 weeks prior to application and continued till day 21 of lactation^(9)^. When the mothers took 100 mg/kg HP, hepatocyte cell vacuolization was determined in the liver of fetuses and 1000 mg/kg treatment increased hepatocellular damage with hyaline degeneration, lobular fibrosis, and disorganization of hepatocytes arrays^(9)^. Their study supports our histologic findings in a great measure. HP showed an inflammatory reaction in the fetal liver tissue in both treated groups. Additionally, 300 mg developed focal necrosis, and hydropic and vacuolar degeneration of fetal liver tissues. Hematopoiesis was not disrupted and continued in the fetal period, and no fatty change in the liver was seen. The same paper also proved that glomerular size was reduced, the Bowman capsule was absent, and that hyaline tubular degeneration developed. Interestingly, these findings were also found in the offspring, even when they were only exposed to HP during the 21-day lactation period^(9)^. Similar to that study, we determined that the diameter of glomeruli was decreased, the Bowman capsule distance was absent, and intense congestion was observed equally in the offspring of both plant-treated groups. Additionally, we determined hydropic and hyaline degeneration in kidney tubules. The structural changes that we detected in the liver and kidney were probably due to free oxygen radical generation and consequently to oxidative damage. There are three different NOS enzyme isoforms: neuronal NOS (nNOS), endothelial NOS (eNOS), and iNOS, which are stimulated by certain cytokines. In pathologic conditions, macrophages and smooth muscle cells in hepatocytes induce iNOS and produce nitric oxide (NO). Excess production of NO results in oxidative tissue damage. Immunohistochemically, we applied iNOS antibody staining to detect the presence of oxidative damage. We demonstrated that the HP produced oxidative damage in the liver and kidney tissues of the fetuses.

### Study Limitations

The limitation of this study is HP effects on the other organs such as neuro-development could not been detected, further studies should be performed about safety of HP.

## Conclusion

HP exposure before conception diminished the pregnancy rate and decreased the fetal number; during pregnancy it tented to increase the duration of gestation, and deteriorated fetal development as determined through body weight. It also damaged liver and kidney tissues, most probably due to oxidative stress at both doses, as supported with iNOS antibody staining. Therefore, HP should not be recommended to any women who want to be pregnant or who are pregnant, because it can be harmful for both fetal and maternal health.

## Figures and Tables

**Table 1:**
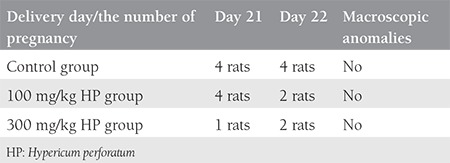
Morphologic evaluations of the control group and treated groups

**Table 2:**
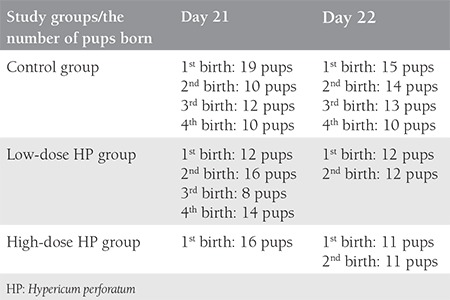
Number of pups in all groups

**Table 3:**
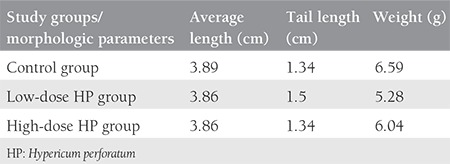
Morphologic evaluations of the offspring in all groups
